# Overexpression of Mycothiol Disulfide Reductase Enhances *Corynebacterium glutamicum* Robustness by Modulating Cellular Redox Homeostasis and Antioxidant Proteins under Oxidative Stress

**DOI:** 10.1038/srep29491

**Published:** 2016-07-07

**Authors:** Meiru Si, Chao Zhao, Bing Zhang, Dawei Wei, Keqi Chen, Xu Yang, He Xiao, Xihui Shen

**Affiliations:** 1 State Key Laboratory of Crop Stress Biology for Arid Areas and College of Life Sciences, Northwest A&F University, Yangling, Shaanxi 712100, China; 2 College of Plant Protection, Northwest A&F University, Yangling, Shaanxi 712100, China; 3 College of Life Sciences, Qufu Normal University, Qufu, Shandong 273165, China

## Abstract

Mycothiol (MSH) is the dominant low-molecular-weight thiol (LMWT) unique to high-(G+C)-content Gram-positive *Actinobacteria*, such as *Corynebacterium glutamicum*, and is oxidised into its disulfide form mycothiol disulfide (MSSM) under oxidative conditions. Mycothiol disulfide reductase (Mtr), an NADPH-dependent enzyme, reduces MSSM to MSH, thus maintaining intracellular redox homeostasis. In this study, a recombinant plasmid was constructed to overexpress Mtr in *C. glutamicum* using the expression vector pXMJ19-His_6_. Mtr-overexpressing *C. glutamicum* cells showed increased tolerance to ROS induced by oxidants, bactericidal antibiotics, alkylating agents, and heavy metals. The physiological roles of Mtr in resistance to oxidative stresses were corroborated by decreased ROS levels, reduced carbonylation damage, decreased loss of reduced protein thiols, and a massive increase in the levels of reversible protein thiols in Mtr-overexpressing cells exposed to stressful conditions. Moreover, overexpression of Mtr caused a marked increase in the ratio of reduced to oxidised mycothiol (MSH:MSSM), and significantly enhanced the activities of a variety of antioxidant enzymes, including mycothiol peroxidase (MPx), mycoredoxin 1 (Mrx1), thioredoxin 1 (Trx1), and methionine sulfoxide reductase A (MsrA). Taken together, these results indicate that the Mtr protein functions in *C. glutamicum* by protecting cells against oxidative stress.

Aerobic organisms unavoidably encounter reactive oxygen species (ROS) including hydrogen peroxide (H_2_O_2_), the superoxide anion (O_2_·^−^), alkyl hydroperoxide (ROOH; cumene hydroperoxide and *t*-butyl hydroperoxide), and the hydroxyl radical (HO.) during aerobic metabolism or following exposure to unfavourable conditions that trigger the production of ROS^1^,^2^. Excess ROS leads to oxidative stress and subsequently damages a wide range of biological molecules including DNA, lipids and carbohydrates, ultimately resulting in cellular damage^3^. To cope with the adverse effects of ROS, enzymatic and non-enzymatic antioxidant systems have evolved^4^. Increasing reports show that sulfhydryl groups (-SH) play a key role in the response to oxidative stress and particularly in antioxidant systems involved in maintaining the redox homeostasis of cells^5^.

Low-molecular-weight (LMW) thiols, which belong to the non-enzymatic systems, act as redox buffers and are essential for the cellular defence against ROS, thus maintaining the reducing state of the cytoplasm. Eukaryotes and Gram-negative bacteria produce the tripeptide glutathione (GSH; γ-L-glutamyl-L-cysteinylglycine) as their LMW thiol redox buffer^6^,^7^, whereas the Gram-positive phylum *Actinobacteria*, such as *Mycobacterium tuberculosis*, *Mycobacterium smegmatis*, and *Corynebacterium glutamicum*, produces the related redox buffer mycothiol (MSH; AcCys-GlcN-Ins)^8^,^9^. Like GSH, MSH plays important roles in protection of the cell against oxidative stress and detoxification of various xenobiotics (Fig. 1)^10^,^11^,^12^,^13^,^14^,^15^,^16^,^17^,^18^,^19^,^20^,^21^,^22^. The redox-active sulfhydryl group of MSH protects cells from ROS by directly scavenging free radicals and by serving as a cofactor for antioxidant enzymes such as mycothiol peroxidase (MPx) and methionine sulfoxide reductase A (MsrA), in conjunction with mycoredoxin (Mrx1)^12^,^13^. MSH detoxifies incoming xenobiotics such as alkylating agents, electrophiles, and antibiotics, by forming MSH *S*-conjugates (MS-R, where R is the toxin)^14^. These *S*-conjugates are subsequently cleaved by the amidase Mca, resulting in the mercapturic acid derivative, AcCysR, and glucosaminylinositol, GlcN-Ins, which is recycled back to mycothiol^14^. MSH is also an essential cofactor for maleylpyruvate isomerase^19^,^20^,^21^, alcohol dehydrogenase MscR^16^,^22^, formaldehyde dehydrogenase AdhE^15^,^22^, and arsenate reductase^17^. Recently, protein *S*-mycothiolation, a reversible post-translational thiol-modification, was discovered as an important thiol protection mechanism that protects active-site cysteine residues of key enzymes against irreversible overoxidation to sulfonic acids^18^.

When subjected to oxidative stress, MSH is oxidised to mycothione (mycothiol disulfide; MSSM), a dimer of two MSH molecules linked by a disulfide bond. To maintain high levels of MSH in the cell, MSSM is reduced back to MSH by an NADPH-dependent mycothiol disulfide reductase (Mtr)^23^. Identified by homology to glutathione reductase, Mtr is a homodimeric flavoprotein disulfide isomerase containing a redox-active disulfide involved in substrate disulfide reduction, and requiring FAD as a cofactor^24^,^25^,^26^. Mtr appears to be essential for the viability of *M. tuberculosis* but not *M. smegmatis*^23^. Although many other MSH-related antioxidative enzymes, such as MPx, MsrA, Mrx1, and Mca, have been investigated extensively, much less is known about Mtr^12^,^13^,^17^,^27^,^28^,^29^. However, several studies have suggested the importance of Mtr for *C. glutamicum*, a model organism of the high-(G+C)-content Gram-positive *Actinobacteria*, under stress conditions. In a proteome analysis, Fanous *et al*. revealed that *C. glutamicum mtr* was upregulated under cadmium stress^30^. Higher expression of *mtr* was also observed in the deletion mutant of *rshA*, which codes for an anti-sigma factor controlling the function of the stress-responsive extracytoplasmic function-sigma (ECF-σ) factor SigH in *C. glutamicum*^31^. In this study, we systematically examined the physiological roles of Mtr in response to oxidative stress by overexpressing the *mtr* gene in *C. glutamicum*. We present the first evidence that *C. glutamicum* Mtr protects against the damaging effects of ROS induced by various exogenous oxidative stresses via modulating the activity of antioxidant proteins and the MSH:MSSM ratio.

## Results

### Overexpression of *mtr* promotes resistance to oxidants, alkylating agents, bactericidal antibiotics, and heavy metals

To assess the physiological roles of Mtr in *C. glutamicum*, we attempted to construct an *mtr*-null mutant. Unfortunately, consistent with reports that *mtr* is an essential gene in *M. tuberculosis*^32^, no viable colonies were obtained after multiple attempts to knock out *mtr*. Thus, to evaluate the physiological roles of Mtr, we constructed a *C. glutamicum* strain overexpressing *mtr* by introducing the expression plasmid pXMJ19-His_6_-*mtr*. The expression of the *mtr* gene was confirmed by SDS-PAGE and western blot analysis (Fig. S1).

**Figure 2:**
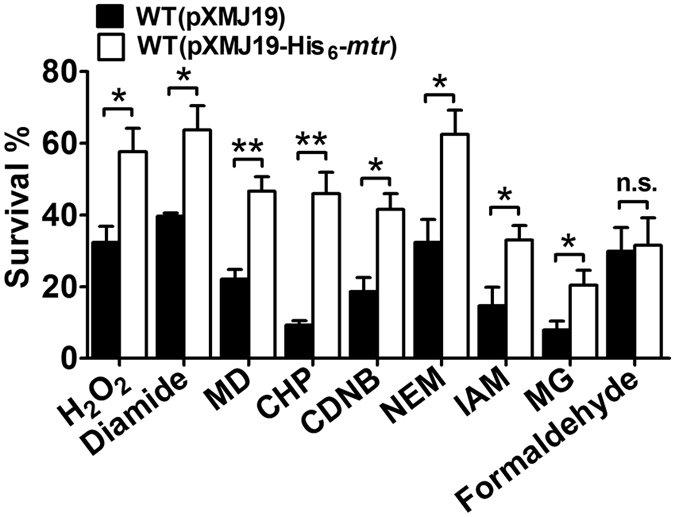
Effects of Mtr overexpression on oxidants and alkylating agents-induced oxidative stresses resistance in *C. glutamicum*. Survival of the *C. glutamicum* WT(pXMJ19) and WT(pXMJ19-His_6_-*mtr*) strains after challenging with various oxidants including H_2_O_2_ (100 mM), diamide (10 mM), CHP (11 mM), MD (4 mM), and formaldehyde (20 mM), and different alkylating agents containing CDNB (70 mM), NEM (16 mM), IAM (40 mM), and MG (10 mM) for 30 min. Mean values with standard deviations (error bars) from at least three independent experiments are shown. n.s.: not significant. *P ≤ 0.05. **P ≤ 0.01.

To investigate whether Mtr participates in protection against various oxidants in *C. glutamicum*, we examined the sensitivities of the overexpressing strain WT(pXMJ19-His_6_-*mtr*) and the vector only control WT(pXMJ19). As shown in Fig. 2, overexpression of *mtr* significantly increased the resistance of wild-type *C. glutamicum* to various oxidants, such as H_2_O_2_, diamide, cumene hydroperoxide (CHP), and menadione (MD), compared to the vector-only control. However, overexpression of *mtr* did not improve the resistance of *C. glutamicum* to the reductant formaldehyde, which is cleared from the cells via a MSH-dependent formaldehyde dehydrogenase but independently of Mtr^15^. These data indicate that Mtr is critical for protection against oxidants that induce the formation of ROS.

It was shown previously that exposure of bacteria to alkylating agents causes excess formation of ROS and subsequently leads to oxidative stress^33^. Thus, we were prompted to examine whether elevated *mtr* levels play a role in resisting alkylating agents in *C. glutamicum*. Overexpressing *mtr* enhanced the robustness of *C. glutamicum* against various alkylating agents, with the observation that the WT(pXMJ19-His_6_-*mtr*) strain was more resistant to 1-chloro-2,4-dinitrobenzene (CDNB), *N*-ethylmaleimide (NEM), iodoacetamide (IAM), and methylglyoxal (MG) than the WT(pXMJ19) control strain, showing 1.40-, 1.66-, 1.42-, and 1.73-fold increases in survival rate, respectively (Fig. 2).

**Figure 3:**
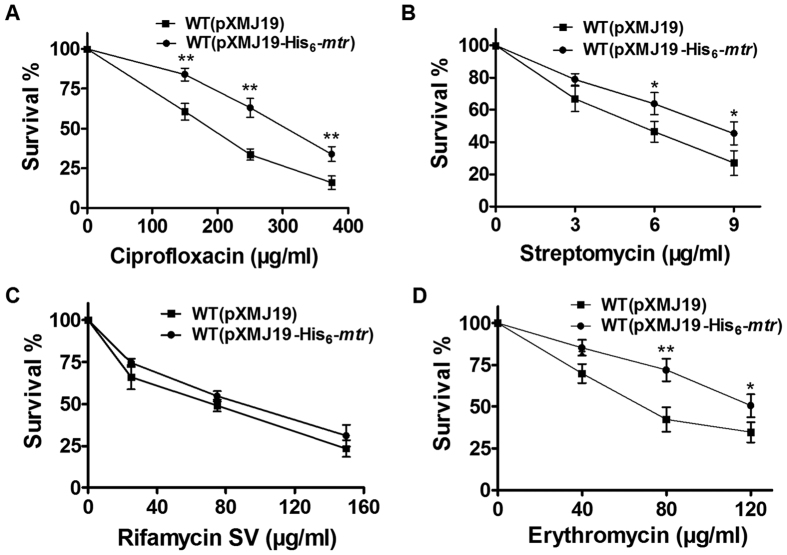
Survival of the *C. glutamicum* WT(pXMJ19-His_6_-*mtr*) and WT(pXMJ19) strains after challenging with different antibiotics. Survival of the *C. glutamicum* WT(pXMJ19) and WT(pXMJ19-His_6_-*mtr*) strains after challenging with different concentrations of ciprofloxacin, streptomycin, rifamycin SV, and erythromycin for 1 h. Mean values with standard deviations (error bars) from at least three independent experiments are shown. *P ≤ 0.05. **P ≤ 0.01.

Bactericidal antibiotics can contribute to oxidative cellular conditions through a common mechanism: inducing ROS formation^34^. To examine the impact of *mtr* overexpression on resistance to bactericidal antibiotics, the cellular viability of WT(pXMJ19) and WT(pXMJ19-His_6_-*mtr*) was tested in LB medium containing different bactericidal antibiotics at various concentrations. As shown in Fig. 3, for streptomycin, erythromycin, and ciprofloxacin, significant growth inhibition was observed in the vector-only control WT(pXMJ19). However, WT(pXMJ19-His_6_-*mtr*) showed significantly higher survival rates when exposed to these substances. Notably, as a control, no significant difference in survival rate was observed between WT(pXMJ19) and WT(pXMJ19-His_6_-*mtr*) treated with the bacteriostatic antibiotic rifamycin SV, which does not stimulate ROS production^34^.

**Figure 4:**
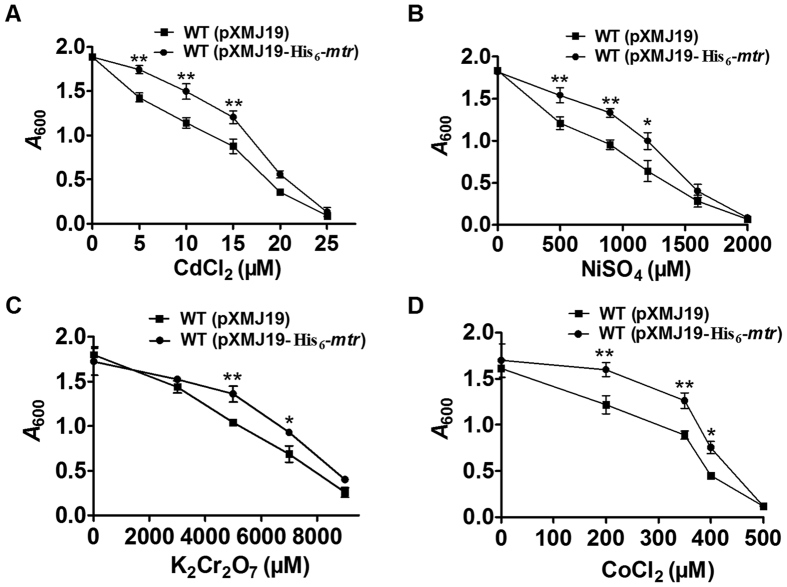
Sensitivity assays of the *C. glutamicum* WT(pXMJ19-His_6_-*mtr*) and WT(pXMJ19) strains against heavy metals. (**A–D**) Growth (*A*_600_) of the *C. glutamicum* WT(pXMJ19) and WT(pXMJ19-His_6_-*mtr*) strains after 24 h at 30 °C in LB medium containing increasing concentrations of Cd^2+^ (**A**), Ni^2+^ (**B**), Cr^6+^ (**C**), and Co^2+^ (**D**) was recorded. Mean values with standard deviations (error bars) from at least three independent experiments are shown. *P ≤ 0.05. **P ≤ 0.01.

Heavy metals can enhance the production of ROS and induce oxidative stress when taken up in excessive amounts, subsequently resulting in imbalanced redox state, protein peroxidation, and biomolecule damage^2^. To determine whether Mtr also protects *C. glutamicum* from heavy metal toxicity, growth of WT(pXMJ19) and WT(pXMJ19-His_6_-*mtr*) in LB medium containing various concentrations of heavy metals was examined. As shown in Fig. 4, in conditions challenged with the heavy metals CdCl_2_, NiSO_4_, K_2_Cr_2_O_7_, and CoCl_2_, WT(pXMJ19-His_6_-*mtr*) grew significantly better than WT(pXMJ19). These data suggest that overexpression of Mtr in wild-type *C. glutamicum* enhances its resistance to heavy metal stress.

These findings demonstrate that Mtr plays important roles in protecting *C. glutamicum* against ROS-inducing oxidants, alkylating agents, bactericidal antibiotics, and heavy metal stress. However, the effect of Mtr overexpression on oxidative stress resistance was almost completely abolished in the MSH-null Δ*mshC* background (Fig. S2), indicating a direct linkage between the antioxidant activities of Mtr and MSH.

### Mtr is able to reduce ROS levels under stress conditions

**Figure 5:**
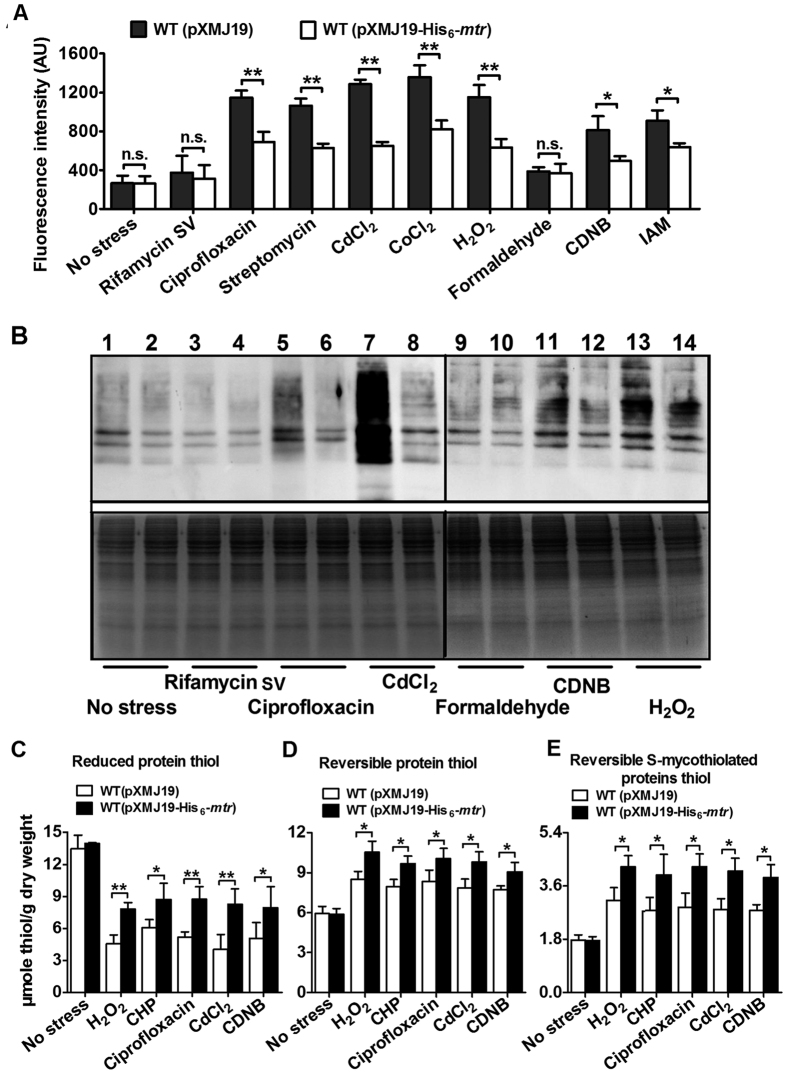
Overexpression of Mtr reduces ROS production under oxidative stress conditions. (**A**) ROS levels in *C. glutamicum* strains expressing Mtr were measured using the DCFHDA fluorescence determination assay after exposure to indicated reagents. Bars represent the fluorescence intensity in arbitrary units (AU). Mean values with standard deviations (error bars) from at least three independent experiments are shown. n.s.: not significant. *P ≤ 0.05. **P ≤ 0.01. (**B**) Protein carbonyl contents were analysed by Western blotting with an anti-DNPH antibody after exposure to various stressors for 30 or 60 min at 30 °C. A parallel run stained with Coomassie Brilliant Blue is shown in the bottom panel. Total proteins were extracted from vector-expressing (lanes 1, 3, 5, 7, 9, 11, and 13) and *mtr*-expressing (lanes 2, 4, 6, 8, 10, 12, and 14) *C. glutamicum* cells. (**C–E**) Analysis of protein thiols. Proteins were extracted from WT(pXMJ19) and WT(pXMJ19-His_6_-*mtr*) cells before and after exposure to multiple stressors. Reduced protein thiols (**C**), reversible protein thiols (**D**), and reversible *S*-mycothiolated proteins thiols (**E**) were quantified using DTNB as described in “Methods”. Mean values with standard deviations (error bars) from at least three independent experiments are shown. *P ≤ 0.05. **P ≤ 0.01.

Because MSH is a key nonenzymatic antioxidant that protects cells from ROS damage by directly scavenging free radicals and by serving as a cofactor for antioxidant enzymes such as MPx and MsrA, we were prompted to investigate whether overexpression of Mtr is able to reduce ROS levels under oxidative stress conditions. Intracellular ROS levels were assessed fluorometrically with 2′, 7′-dichlorodihydrofluorescein diacetate (DCFHDA). As shown in Fig. 5A, the WT(pXMJ19-His_6_-*mtr*) strain had significantly lower levels of ROS compared to the WT(pXMJ19) strain after exposure to ciprofloxacin (375 μg/ml), streptomycin (9 μg /ml), CdCl_2_ (300 μM), CoCl_2_ (7.5 mM), H_2_O_2_ (100 mM), CDNB (70 mM) and IAM (40 mM). For example, following exposure to H_2_O_2_ (100 mM), 0.66-fold higher levels of ROS were observed in WT(pXMJ19) cells compared to WT(pXMJ19-His_6_-*mtr*) cells. However, no significant difference in ROS levels was observed in WT(pXMJ19-His_6_-*mtr*) exposed to rifamycin SV and formaldehyde compared to the vector-only control (Fig. 5A). Moreover, for both WT(pXMJ19-His_6_-*mtr*) and WT(pXMJ19), there was no significant difference in ROS levels between rifamycin SV- or formaldehyde-treated strains and untreated strains, indicating that the two agents did not induce ROS production. These data indicate that Mtr is involved in the reduction of ROS induced by multiple oxidative stressors in *C. glutamicum*.

ROS escaping from the antioxidant defence system are more apt to react with the cysteine thiol groups of proteins, which results in reversible inter- or intra-protein disulfides (PrSSPr, PrSSPr), and mixed disulfides with LMW thiols; irreversible sulfoxidation products and carbonylation^35^,^36^. To test whether Mtr functions in protecting against protein carbonylation under oxidative stress conditions, we isolated total proteins from the *mtr*-overexpressing WT(pXMJ19-His_6_-*mtr*) and the vector only control WT(pXMJ19) grown in the presence of different stressors. Carbonyl groups in the proteins were derivatised with 2, 4-dinitrophenylhydrazine (DNPH) and detected by western blot using an anti-DNPH antibody. As shown in Fig. 5B, rifamycin SV treatment caused background-level carbonylation, similar to that of the no-stress control. Similarly, treatment with the formaldehyde, which does not induce ROS production, also caused background-level carbonylation. However, treatment with ROS-generating agents, such as ciprofloxacin, CdCl_2_, CDNB, and H_2_O_2_, caused significantly more carbonylation than no-stress treatment in the vector-only control strain WT(pXMJ19). Notably, WT(pXMJ19-His_6_-*mtr*) cells overexpressing *mtr* showed significantly lower carbonyl contents than WT(pXMJ19) cells under treatment with these ROS-generating agents. However, there was no significant difference in carbonyl contents between WT(pXMJ19) cells and WT(pXMJ19-His_6_-*mtr*) cells treated with rifamycin SV and formaldehyde. These results clearly demonstrate that, under oxidative stress, *mtr* overexpression reduced intracellular ROS levels and thus protein carbonylation caused by ROS.

Reversible thiol-oxidation such as inter- or intra-protein disulfides and mixed disulfides with LMW thiols occurred mainly under conditions of moderate oxidative stress^37^. Since the Mrx1/MSH/Mtr pathway regulates the level of protein *S*-mycothiolation and other Cys oxidations, the level of reduced protein thiols would be expected to be increased under oxidative stress when Mtr is overexpressed. To test this hypothesis, we determined the level of reversible thiol-oxidation in the Mtr-overexpressing strain under various oxidative stresses. As shown in Fig. 5C, loss of reduced protein thiols was detected in both WT(pXMJ19) and WT(pXMJ19-His_6_-*mtr*) treated with ROS-inducing agents. However, compared to WT(pXMJ19), the magnitude of the decrease was less in WT(pXMJ19-His_6_-*mtr*) in the presence of various agents, suggesting that overexpression of Mtr protected or regenerated reduced protein thiols. In contrast, the level of reversible thiol-oxidation in WT(pXMJ19-His_6_-*mtr*) was higher than that in WT(pXMJ19) (Fig. 5D), suggesting an important role for Mtr in protecting protein thiols. Overexpression of Mtr also increased the generation of *S*-mycothiolated proteins (protein-SSM) under oxidative stress (Fig. 5E).

### Overexpression of Mtr increases the MSH:MSSM ratio under stress conditions

**Figure 6:**
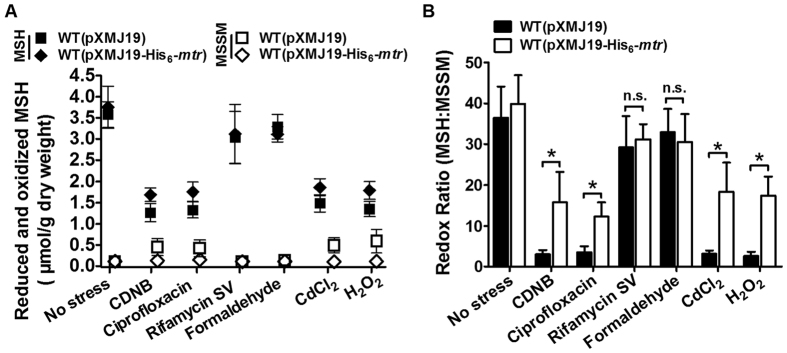
MSH and MSSM levels in WT(pXMJ19-His_6_-*mtr*) and WT(pXMJ19) strains upon treatment with different stressors. (**A**) MSH (reduced form) in WT(pXMJ19-His_6_-*mtr*) (closed rhombus) and WT(pXMJ19) (closed squares), and MSSH (oxidised form) in WT(pXMJ19-His_6_-*mtr*) (open rhombus) and WT(pXMJ19) (open squares). Mean values with standard deviations (error bars) from at least three independent experiments are shown. (**B**) The redox ratios of MSH:MSSM in WT(pXMJ19-His_6_-*mtr*) and WT(pXMJ19) strains upon exposure to different stressors. Mean values with standard deviations (error bars) from at least three independent experiments are shown. n.s.: not significant. *P ≤ 0.05.

To investigate whether the decreased ROS levels in Mtr-overexpressing cells were attributable to the reduced MSSM levels and increased MSH:MSSM ratio, we analysed cellular MSH and MSSM levels. The results in Fig. 6A demonstrated that MSH was depleted rapidly in WT(pXMJ19) and WT(pXMJ19-His_6_-*mtr*) strains by ROS-inducing exogenous agents within 30 min. Accompanying the rapid drop in MSH levels, an obvious increase in MSSM levels was observed in CDNB-, ciprofloxacin-, CdCl_2_-, and H_2_O_2_-treated WT(pXMJ19) strains; little increase was observed in WT(pXMJ19-His_6_-*mtr*) treated with the above-mentioned agents. In contrast, upon treatment with formaldehyde and rifamycin SV, no excess MSSM generation or MSH consumption was observed in both WT(pXMJ19) and WT(pXMJ19-His_6_-*mtr*). It is noteworthy that the MSH levels of WT(pXMJ19-His_6_-*mtr*) were higher than those of WT(pXMJ19) under H_2_O_2_, ciprofloxacin, CdCl_2_, and CDNB stresses. Moreover, the MSSM levels of WT(pXMJ19-His_6_-*mtr*) cells were lower than those of WT(pXMJ19) cells under H_2_O_2_, ciprofloxacin, CdCl_2_, and CDNB stresses. However, we did not observe any significant difference in MSH or MSSM levels when the WT(pXMJ19) and WT(pXMJ19-His_6_-*mtr*) strains were exposed to formaldehyde and rifamycin SV.

Next, the MSH:MSSM redox ratios of WT(pXMJ19) and WT(pXMJ19-His_6_-*mtr*) under different exogenous agent treatments were calculated. As shown in Fig. 6B, the MSH:MSSM redox ratios of WT(pXMJ19-His_6_-*mtr*) under H_2_O_2_, ciprofloxacin, CdCl_2_, and CDNB stresses were obviously higher than those of the WT(pXMJ19). However, there was no significant difference in the MSH:MSSM redox ratios between WT(pXMJ19) and WT(pXMJ19-His_6_-*mtr*) under formaldehyde and rifamycin SV stresses. The high MSH:MSSM redox ratios of WT(pXMJ19-His_6_-*mtr*) treatment with various exogenous agents indicated that Mtr plays an important role in maintaining intracellular redox homeostasis.

Moreover, in WT(pXMJ19-His_6_-*mtr*) and WT(pXMJ19) cells under stress, the sum of reduced MSH and MSSM levels was lower than that in the corresponding untreated strains, as reported by Pöther *et al*. for *Bacillus subtilis* and *Staphylococcus aureus*^38^. These data, together with the enhanced protein-SSM generation under oxidative stress (Fig. 5E), indicate that the drastic depletion of the total reduced MSH and MSSM levels led to an increase in *S*-mycothiolation of protein thiols.

### Overexpression of *mtr* enhances the activity of antioxidant enzymes

**Figure 7:**
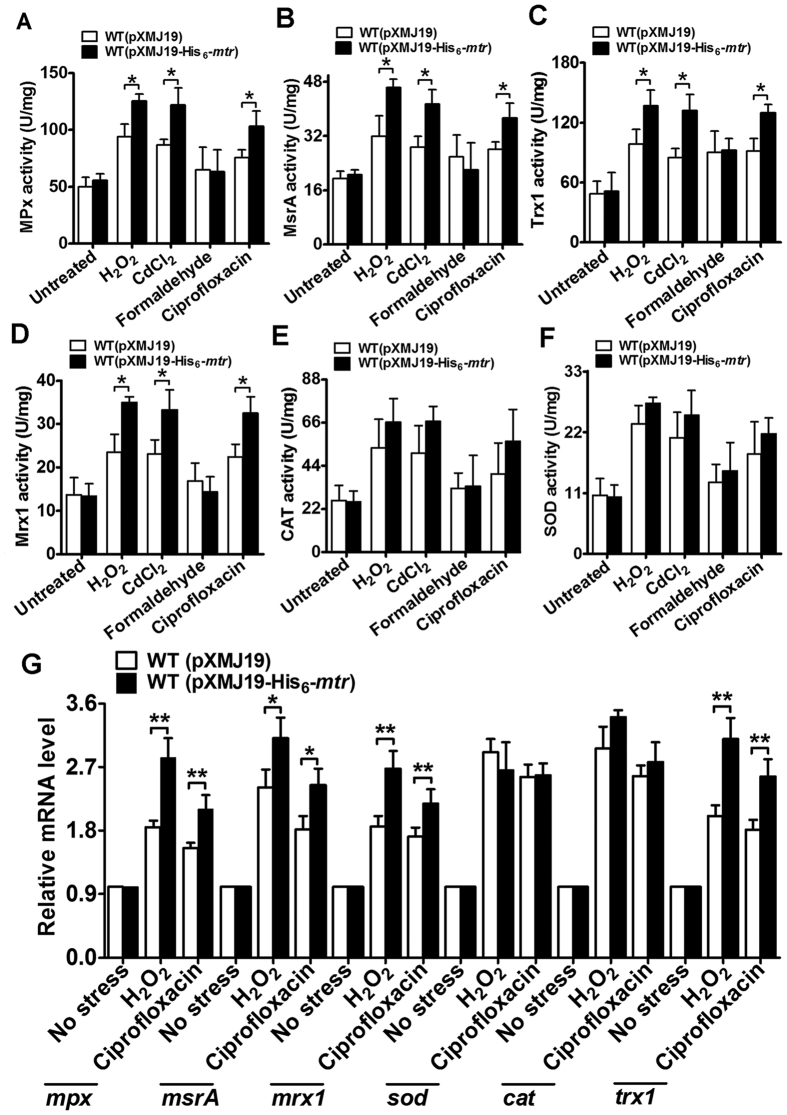
Activity and expression level of antioxidant enzymes. (**A–F**) Activities of antioxidative enzymes. Bacterial cells grown to stationary phase were exposed to 100 mM H_2_O_2_, 20 mM formaldehyde, 0.3 mM CdCl_2_, 70 mM CDNB, and 375 μg/ml ciprofloxacin for 30 or 60 min at 30 °C. Crude protein extracts were prepared and then used to measure enzyme activity. Each enzyme activity is represented as U/mg of protein. Mean values with standard deviations (error bars) from at least three independent experiments are shown. *P ≤ 0.05. (**G**) The WT(pXMJ19) and WT(pXMJ19-His_6_-*mtr*) strains grown to mid-log phase were exposed to multiple stressors for 30 min at 30 °C. The levels of indicated gene expression were determined by qRT-PCR. The mRNA levels were presented relative to the value obtained from cells without treatment. The values represent the mean results from three independent cultivations, with standard errors. *P ≤ 0.05. **P ≤ 0.01. CAT, catalase; SOD, superoxide dismutase; MPx, mycothione peroxidase; Mrx1, mycoredoxin 1; MsrA, methionine sulfoxide reductase A; Trx1, thioredoxin 1.

Overexpression of a glutathione reductase (GR) from *Brassica rapa* in *Escherichia coli* enhanced cellular redox homeostasis by provoking the expression of a variety of antioxidant enzymes, including catalase (CAT), superoxide dismutase (SOD), glutathione peroxidase (Gpx), and glucose-6-phosphate dehydrogenase (G6PDH)^7^. To explore whether overexpression of Mtr has the same effect, we measured the activities of six key antioxidant enzymes (CAT, SOD, MPx, MsrA, Mrx1, and Trx1) in *C. glutamicum* treated with various stressors. As seen in Fig. 7A–D, the activities of MPx, MsrA, Mrx1, and Trx1 were significantly higher in WT(pXMJ19-His_6_-*mtr*) cells than in WT(pXMJ19) cells under H_2_O_2_, CdCl_2_, and ciprofloxacin stress. However, there was no significant difference in the activities of MPx, Mrx1, MsrA, and Trx1 between the WT(pXMJ19) and WT(pXMJ19-His_6_-*mtr*) extracts under formaldehyde stress. Although the CAT and SOD activity under exposition were increased in both WT(pXMJ19-His_6_-*mtr*) and WT(pXMJ19) cells, WT(pXMJ19-His_6_-*mtr*) cells did not exhibit a significant higher CAT and SOD activity in the presence of stressor compare to WT(pXMJ19) cells (Fig. 7E,F). This indicates that overexpression of Mtr had a marginal effect on CAT and SOD activity.

To determine whether increased enzyme expression levels accompanied the increased antioxidant enzyme activities, we performed qRT-PCR analysis. As shown in Fig. 7G, although the expression of all six antioxidant enzymes under exposition were increased in both WT(pXMJ19-His_6_-*mtr*) and WT(pXMJ19) cells, *mpx*, *msrA*, *mrx1*, and *trx1* expression in WT(pXMJ19-His_6_-*mtr*) cells was obviously higher than in WT(pXMJ19) cells under oxidative stress, suggesting that Mtr overexpression enhances the expression of antioxidant enzymes associated with MSH and Mtr. Consistent with the finding that overexpression of Mtr had a marginal effect on CAT and SOD activity, there was no significant difference in the *cat* and *sod* expression levels of WT(pXMJ19-His_6_-*mtr*) and WT(pXMJ19) cells under oxidative stress (Fig. 7G).

### Induction of *mtr* expression by multiple stressors and its positive regulation by SigH

**Figure 8:**
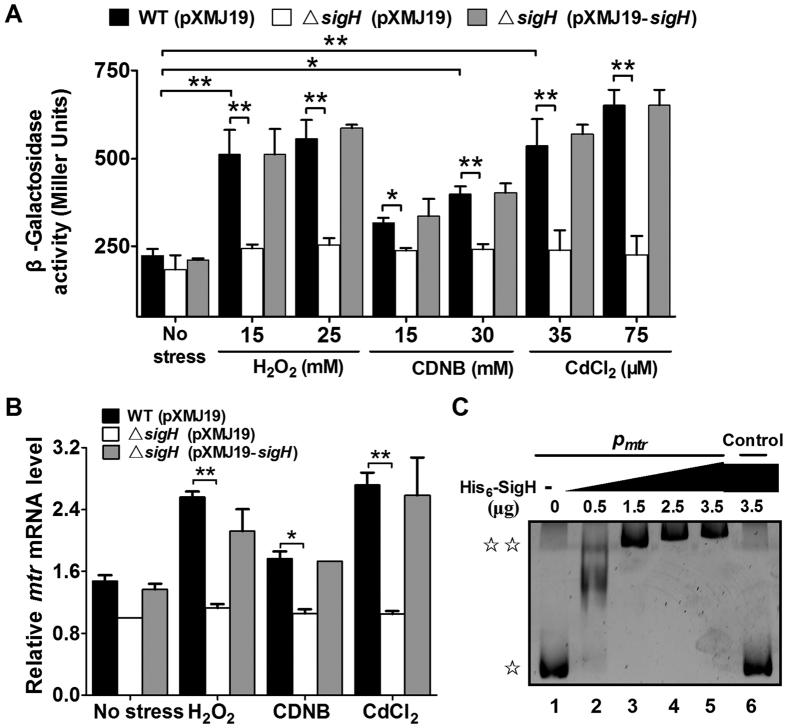
Positive regulation of *C. glutamicum mtr* expression by SigH. (**A**) β-Galactosidase analysis of the *mtr* promoter activity was performed using the transcriptional *P*_*mtr*_::lacZ chromosomal fusion reporter expressed in the wild-type, Δ*sigH* mutant, and complemented strain Δ*sigH*(pXMJ19-*sigH*). Exponentially growing *C. glutamicum* cells (100 μl) induced with different toxic agents at the indicated concentrations for 30 min were added to the enzyme reaction system. β-Galactosidase activity was assayed as described in “Methods.” Mean values with standard deviations (error bars) from at least three repeats are shown. *P ≤ 0.05. **P ≤ 0.01. (**B**) qRT-PCR revealed that expression of *mtr* was under strict positive regulation by SigH. Exponentially growing *C. glutamicum* cells were exposed to different toxic agents at the indicated concentrations for 30 min. Levels of *mtr* expression were determined by qRT-PCR. The mRNA levels are presented relative to the value obtained from wild-type cells without treatment. Mean values with standard deviations (error bars) from at least three repeats are shown. *P ≤ 0.05. **P ≤ 0.01. (**C**) Interactions between SigH and the *mtr* promoter were analysed by EMSA. Increasing amounts of SigH used were 0, 0.5, 1.5, 3.0, and 3.5 μg. As a negative control, a 400-bp fragment from the *mtr* coding region amplified with primers Control-F and Control-R, replacing the 400-bp *mtr* promoter, was incubated with 3.5 μg of His_6_-SigH in the binding assay. (☆) free DNA and (☆☆) major DNA-protein complex.

We have shown that Mtr is involved in protecting *C. glutamicum* against multiple stressors. In the next stage, we performed qRT-PCR and LacZ activity profiling to examine whether *mtr* expression responds to multiple stress inducers at the transcriptional level. The LacZ activity of the *P*_*mtr*_::*lacZ* chromosomal promoter fusion reporter in the *C. glutamicum* wild-type was quantitatively measured in bacterial cells either untreated or treated with various stressors at different concentrations (Fig. 8A). The level of *mtr* expression was increased by approximately 2.48-, 2.91-, and 1.78-fold in the reporter strain treated with H_2_O_2_, CdCl_2_, and CDNB, respectively, compared to untreated samples (Fig. 8A). A similar pattern of *mtr* expression in response to oxidative stress was also observed by qRT-PCR analysis (Fig. 8B). These results clearly demonstrate that oxidative stress induces *mtr* expression, which in turn directly contributes to the tolerance of *C. glutamicum* to oxidative stress conditions.

Recently, a transcriptomics study reported increased expression of *mtr* in a mutant of *rshA*, which encodes an anti-sigma factor that controls the function of SigH in *C. glutamicum*^31^. As SigH, the stress-responsive extracytoplasmic function-sigma (ECF-σ) factor, was reported to respond to thiol-oxidative stress and regulate the expression of multiple resistance genes, we were prompted to examine whether *mtr* expression was subjected to SigH regulation by measuring the transcription of chromosomal *P*_*mtr*_::*lacZ* fusions. A significant decrease in LacZ activity was observed for the exponentially grown Δ*sigH*(pXMJ19) mutant exposed to H_2_O_2_, CdCl_2_, and CDNB for 30 min, compared to the wild-type (Fig. 8A). The reduction in *mtr* expression in the Δ*sigH*(pXMJ19) mutant was almost fully recovered in the complemented strain Δ*sigH*(pXMJ19-*sigH*) under both oxidative stressor-inducible and non-inducible conditions (Fig. 8A). SigH-dependent *mtr* activation was also confirmed by qRT-PCR analysis (Fig. 8B). These data indicate that the expression of Mtr is SigH-dependent in the presence of the substances tested.

To determine whether SigH regulates *mtr* expression directly, we examined the interaction between His_6_-SigH and the *mtr* promoter by electrophoretic mobility shift assay (EMSA). Incubation of His_6_-SigH with a 400-bp DNA probe harbouring the *mtr* promoter (*P*_*mtr*_) sequence led to retarded mobility of the probe, and the DNA-protein complexes increased in response to increased His_6_-SigH in the reaction (Fig. 8C), indicating direct binding of this protein to the *mtr* promoter. A 400-bp control DNA fragment amplified from the *mtr* coding region showed no detectable His_6_-SigH binding (Fig. 8C). Collectively, these results indicate that multiple oxidative stressors induce the expression of *mtr*, which in turn directly contributes to stressor-induced MSSM reduction, ultimately leading to cell tolerance to these adverse stresses.

## Discussion

The mechanisms of *C. glutamicum* resistance to ROS are diverse. On one hand, exposure to multiple ROS-generating stressors induces the redox-sensitive transcriptional regulators, such as SigH, OxyR, RosR, and so on, which in turn induces the production of various enzymes to combat oxidative stress. The SigH regulon includes enzymes of the mycothiol synthesis pathway (*mshC* and *mshB*), a thioredoxin (*trx*), mycothiol disulfide reductase (*mtr*), and an amidase (*mca*), and OxyR regulates the expression of *cat* and *sod*. The abundant LMW antioxidant, MSH, constitutes a redox buffer in the cytoplasm and is considered the main non-enzymatic antioxidant in high-GC Gram-positive bacteria^8^,^9^. The cysteine thiol of MSH can protect cells against ROS by directly clearing ROS, and in cooperation with Mrx1, by functioning to reduce disulfide bonds for antioxidant enzymes such as MPx and MsrA. In this manner, oxidised mycothiol forms the disulfide mycothione (MSSM), which is recycled back to MSH by Mtr, thus maintaining cellular redox homeostasis. To date, little is known about the physiological functions of Mtr.

In this study, we investigated the physiological roles and underlying mechanisms of Mtr in *C. glutamicum* under multiple stresses. We demonstrated the protective role of *C. glutamicum* Mtr against oxidative stresses induced by ROS-generating oxidants, alkylating agents, bactericidal antibiotics, and heavy metal ions (Figs 2, 3, 4). We also demonstrated that the protective effect of Mtr is attributed to its ability to enhance the activity of some antioxidant enzymes (Fig. 7) and largely recovering the redox ratio of MSH:MSSM (Fig. 6B).

The redox balance between the oxidised LWM thiol and the reduced LWM thiol reflects the organism’s ability to withstand fluctuations due to oxidative stress^9^,^39^. Our observations showed that the basal ratio of MSH to MSSM was ~35:1 in *C. glutamicum* under normal conditions, and decreased to less than 8:1 upon treatment with ROS-inducing agents. The WT(pXMJ19-His_6_-*mtr*) strain always showed significantly higher MSH:MSSM ratios compared to the WT(pXMJ19) strain under oxidative stresses, and the MSH:MSSM ratios corresponded to the survival rates of WT(pXMJ19-His_6_-*mtr*) and WT(pXMJ19) treated with different stressors. That is, the higher the MSH:MSSM ratio, the greater the resistance (Fig. S3). This conclusion is further supported by our observations that the recovery of MSH levels in WT(pXMJ19-His_6_-*mtr*) was accompanied by a decrease in oxidised MSH levels and expression of *mtr* in *C. glutamicum* was induced and positively regulated by SigH under oxidative stress caused by H_2_O_2_, CdCl_2_, ciprofloxacin, and CDNB, indicating that Mtr is not completely saturated in this system. This further suggests that ROS induced by oxidants, alkylating agents, bactericidal antibiotics, and heavy metal ions both directly and indirectly causes oxidation of MSH, followed by a reduction of MSSM by Mtr to enhance the resistance of *C. glutamicum* to adverse stress.

Consistent with a previous report that Mtr is essential in *M. tuberculosis*, we failed to delete the *mtr* gene in *C. glutamicum* after multiple attempts. However, although *M. smegmatis* Mtr was highly homologous to *M. tuberculosis* and *C. glutamicum* Mtr (75% and 54% amino acid identity), Holsclaw *et al*. reported that it is not essential in *M. smegmatis*^23^. This contradiction may be explained by the fact that *M. smegmatis* has a higher cellular level of reduced MSH compared to *M. tuberculosis* and *C. glutamicum* (the basal MSH:MSSM ratio of *M. smegmatis* ranges from 200:1 to 1000:1, compared to 50:1 and 35:1 for *M. tuberculosis* and *C. glutamicum*, respectively)^40^,^41^. Upon treatment with H_2_O_2_ and diamide, the redox ratio in *M. smegmatis* was unaltered^40^. In contrast, the MSH:MSSM ratio in *C. glutamicum* decreased to less than 8:1 upon treatment with ROS-inducing agents. Hence, in the absence of Mtr in *M. smegmatis*, either MSSM was reduced by another thiol reductase, or the high level of MSH in this bacterium compensates for the lack of a reducing enzyme^40^.

The robustness of the WT(pXMJ19-His_6_-*mtr*) response may be explained by the fact that WT(pXMJ19-His_6_-*mtr*) has a higher cellular level of reduced MSH compared to WT(pXMJ19), and is thus resistant to the levels of oxidative stress used in our assays. Another protective strategy of Mtr against oxidative stress is an increase in the activity of multiple antioxidant enzymes. *E. coli* cells respond to the redox stress imposed by ROS-generating agents by increasing the expression of different proteins^7^. These proteins include many components of multilevel antioxidant enzymes with inducible functions for ROS scavenging (CAT, SOD, GR, and GPX), G6PDH, protein synthesis, and metabolic pathways^42^. The expression of CAT, SOD, GPX, and G6PDH was shown to be significantly higher in GR-transformed cells than in control cells during MD, CdCl_2_, and ZnCl_2_ stress^7^. Similarly, the activities of antioxidant enzymes MPx, MsrA, Mrx1, and Trx1 were obviously higher in WT(pXMJ19-His_6_-*mtr*) than in WT(pXMJ19) cells under ROS-generating stress, in agreement with studies that Mrx1, MPx, MsrA, and Trx1 were obviously upregulated in WT(pXMJ19-His_6_-*mtr*) upon treatment with ROS-generating agents (Fig. 7G). This result suggests an indirect relationship between ROS and Mtr. ROS are detoxified by the antioxidant enzymes MPx and MsrA to generate MPx-SOH and MsrA-SOH, and then form the *S*-conjugates MPx-SSM and MsrA-SSM with MSH, followed by being attacked through the action of the thiol-disulfide redox enzymes Mrx1 and Trx1 to form Mrx1-SSM and Trx1-SSM and MPx/MsrA, and ultimately, Mrx1-SSM and Trx1-SSM are reduced by Mtr/MSH. However, the expression levels and activities of CAT and SOD in ROS-inducing agent-treated WT(pXMJ19-His_6_-*mtr*) strains was not elevated compared to those in WT(pXMJ19), possibly because CAT and SOD are regulated by OxyR and are not related to SigH and Mtr^43^. This conclusion further indicates that insufficiently reduced MSH limits the activity of MSH-dependent antioxidant enzymes, but the recovery of reduced MSH levels by adequate Mtr reducing MSSM can meet the needs of antioxidant enzymes under oxidative stress. Another possibility is that the recovery of MSH levels by an adequate reduction of MSSM by Mtr gives antioxidant enzymes a suitable cell environment in which to better function.

In summary, we demonstrated the protective role of Mtr in the oxidative tolerance of *C. glutamicum*. Mtr-mediated stress tolerance appears to be involved in cells’ effective adaptation of their stress response system by inducing antioxidant proteins and largely recovering the MSH:MSSM redox ratio.

## Methods

### Bacterial strains and culture conditions

The bacterial strains and plasmids used in this study are listed in Table S1. *C. glutamicum* and *E. coli* strains were cultured in Luria-Bertani (LB) medium as previously reported^20^. The *C. glutamicum* strain RES167 was the parent of all derivatives used in this study. Sensitivity assays for diverse stress were performed as described^13^. All chemicals, oxidants, alkylating agents, and heavy metals were purchased from Solarbio (Beijing, China). All enzymes were purchased from Sigma-Aldrich (St. Louis, MO). All antibiotics were purchased from Gold Biotechnology (Shanghai, China).

### Cloning, expression, and purification of recombinant proteins

For constructing the Δ*mtr* deletion mutant, the plasmid pK18*mobsacB*-Δ*mtr* was generated by overlap PCR and transformed into relevant *C. glutamicum* RES167 by electroporation^44^. Integration of the introduced plasmids into the chromosome by single crossover was selected on BHIS (brain heart infusion supplemented with 0.5 M sorbitol) plates containing 25 μg/ml kanamycin and 40 μg/ml nalidixic acid. For deletion of the target gene, the kanamycin-resistant strains were grown overnight in liquid BHIS and spread on BHIS plates containing 20% sucrose and 40 μg/ml nalidixic acid. Strains growing on this plate were tested for kanamycin sensitivity (Km^S^) by parallel picking on BHIS plates containing nalidixic acid and sucrose. More than 8000 kanamycin-sensitive and sucrose-resistant clones were tested for deletion by PCR, but all checked clones resulted in wild-type situation. The LacZ fusion reporter vector pK18*mobsacB*-*P*_*mtr*_::lacZ was constructed by fusion of the *mtr* promoter to the *lacZY* reporter gene via overlap PCR^44^. The gene encoding Mtr (NCgl1928) was amplified by PCR using *C. glutamicum* RES167 genomic DNA as template. The DNA fragments were digested and subcloned into similar digested pXMJ19-His_6_ to obtain pXMJ19-His_6_-*mtr*. The pXMJ19-His_6_-*mtr* was transferred into *C. glutamicum* by electrotransformation^45^. Expression in *C. glutamicum* was induced by the addition of 0.5 mM isopropyl β-D-1-thiogalactopyranoside (IPTG) and analyzed by immunoblotting using the anti-His antibody. To express and purify His_6_-tagged proteins, recombinant pET28a plasmids were transformed into *E. coli* BL21(DE3) strains. Recombinant proteins were prepared essentially as described by Xu *et al*.^46^. All primers used in this study are listed in Table S1. The fidelity of all constructs was confirmed by DNA sequencing (Sangon Biotech, Shanghai, China).

### Measurement of intracellular ROS levels and determination of cellular protein carbonylation

Fluorescence dye-based intracellular ROS detection was performed using the fluorescent reporter DCFHDA as previously described^47^. Protein carbonylation assays were performed based on the method described by Vinckx *et al*.^48^.

### Antioxidant enzyme activity

Stationary phases cells with and without diverse stress treatment were harvested by centrifugation and then washed with ice-cold 100 mM Tris–HCl, pH 7.0 containing 10% (v/v) glycerol. Cells were disrupted with 1 g zirconia/silica beads (0.1 mM) (Roth, Karlsruhe, Germany) and a Q-BIOgene FastPrep FP120 instrument (Q-BIOgene, Heidelberg, Germany) by ten times for 30-s cycles at a speed of 6.5 m/s. Enzyme activity was determined immediately in the cell-free supernatant after centrifugation. The catalases activities were measured by monitoring the changes of H_2_O_2_ at *A*_240_ as described^49^. The reaction mixtures included 5 μ1 1mg/ml crude enzyme, 50 mM Tris-HCl (pH 7.0), and 10 mM H_2_O_2_. The concentration of H_2_O_2_ was calculated using the molar absorption coefficient of H_2_O_2_ at 240 nm (*ε*_240_) of 43.6 M^−1^·cm^−1^. Catalase activity is expressed in units of mmol of H_2_O_2_ decomposed per mg within 1 min. Activity of SOD was estimated by monitoring the photo reduction of nitroblue tetrazolium (NBT) at 540 nm^50^. SOD unit is the quantity of crude enzyme that hamper 50% photo reduction of NBT and is expressed as U/mg protein within 1min. MPx, MsrA, Mrx1, and Trx1 activity were performed by monitoring the decrease of NADPH at 340 nm as described^12^,^13^,^18^,^51^. The activities of MPx, Mrx1, Trx1, and MsrA were determined using 4 μM thioredoxin reductase (TrxR)/40 μM Trx1 as the electron donor and 1000 μM Linoleic acid hydroperoxides (LA-OOH) as substrates, 1 mM MSH/5 μM Mtr as the electron donor and 1000 μM S-mycothiolated peroxiredoxin (Prx-SSM) as substrates, 5 μM TrxR as the electron donor and 0.32 mM insulin as substrates, 4 μM TrxR/40 μM Trx1 as the electron donor and 100 mM methionine sulfoxide (MetO) as the electron donor, respectively. The reaction mixtures contained 50 mM Tris-HCl buffer (pH 7.5), 1 mM EDTA, 500 μM NADPH, 5 μ1 1mg/ml crude enzyme, the relevant electron donor, and substrates. The number of micromoles of NADPH was calculated using the molar absorption coefficient of NADPH at 340 nm (*ε*_340_) of 6220 M^−1^·cm^−1^. Activity is expressed in units corresponding to 1 μmol NADPH consumption within 1 min per mg crude enzyme. All the above activities were determined after subtracting the spontaneous reduction rate observed in the absence of crude enzyme.

### Analysis of protein thiol levels

Protein thiol levels were assayed based on the method described by Si *et al*.^13^ and Pöther *et al*.^38^. To determine the levels of reduced protein thiols, overnight-grown cultures of *C. glutamicum* exposed to various stressors were harvested and resuspended in 600 μl extraction buffer [50% (vol/vol) acetonitrile (CAN) in 20 mM Tris-HCl; pH 8.0]. The suspension was incubated for 30 min at 60 °C. After centrifugation, the supernatant (500 μl) was mixed with 10 μl of 100 mM 5, 5′-dithiobis-(2-nitrobenoic acid) (DTNB), and continued to incubate for 30 min. The supernatant was diluted after centrifugation, *A*_412_ was measured, and the protein thiol content was calculated.

To quantify reversible protein thiol levels, stressors-treated cells were harvested and the pellets were resuspended in denaturing buffer, consisting of 8 M urea, 1% 3-[(3-cholamidopropyl)-dimethylammonio]-1-propanesulfonate (CHAPS), 1 mM EDTA, 200 mM Tris-HCl (pH 8.0), and 100 mM IAM. Cells were disrupted by the above method described in “antioxidant enzyme activity”. Proteins were precipitated in ice-cold acetone followed by centrifugation. The resulting pellet was washed twice with acetone, dried, and resuspended in denaturing buffer without IAM. All reversible thiol modifications were reduced with Tris-(2-carboxyethyl-)-phosphine (TCEP), and proteins were precipitated by ice-cold acetone to remove unreacted TCEP. Subsequently, proteins were re-dissolved in 100 mM DTNB-containing Tris-HCl buffer (pH 8.0), and the newly formed protein thiols were quantified.

To determine the levels of mycothiolated protein thiols, stressors-treated cells were harvested, resuspended in IAM-containing denaturing buffer, and precipitated in ice-cold acetone as described above. The pellet was resuspended and demycothiolated using 20 μM recombinant Mrx1:C15S in the presence of 1 mM MSH, 250 μM NADPH, and 10 μM Mtr for 30 min at room temperature. Subsequently, proteins were precipitated in ice-cold acetone to remove unreacted MSH, re-dissolved in 100 mM DTNB-containing Tris-HCl buffer, and the newly formed protein thiol content was assayed. Tris-HCl buffer containing 20 μM Mrx1:C15S, 1 mM MSH, 250 μM NADPH, and 10 μM Mtr served as the negative control.

### MSH and MSSM determination

MSH concentration was determined according to Yin *et al*. with minor modifications^52^. In brief, Cell disruption was prepared according to the above method described in “antioxidant enzyme activity”. After centrifugation, the supernatant diluted 500 times was used for MSH qualitative determination. Maleylpyruvate, the substrate for maleylpyruvate isomerase (MDMPI), was freshly prepared by reaction of 120 μM gentisate and purified gentisate-1, 2-dioxygenase (G12D) in 50 mM Tris-HCl (pH8.0) until the *A*_330_ did not change. 5 μl diluted cellular supernatant, MDMPI, and fumarylpyruvate hydrolase (FPH) were added to the resulting mixture containing maleylpyruvate. To detect the presence of MSSM, we modified the assay as follows: first, warm NEM (dissolved in 50% acetonitrile/water and 20 mM HEPES, pH 8.0) was added to bind all thiol groups. Second, cells were broken and cellular debris was pelleted by centrifugation, followed by the addition of β-mercaptoethanol and alcohol to the supernatant. Finally, dithiothreitol (DTT) was added to the resulting supernatant to reduce all disulfide bonds. The reduced MSH molecules were then measured as described above.

### Electrophoretic mobility shift assay (EMSA)

EMSA was performed using the method of Si *et al*.^13^. To reduce non-specific binding, a shorter DNA promoter probe (*P*_*mtr*_; 400 bp) containing the predicted SigH binding site was amplified using primers *P*_*mtr*_-F2 and Mtr-R (Table S1). Increasing concentrations of purified His_6_-SigH (0–4 μg) were incubated with 20 ng of DNA probes in EMSA buffer. After the binding reaction mixture was subjected to electrophoresis on a 6% native polyacrylamide gel, the DNA probe was detected with SYBR Green (Invitrogen, Carlsbad, CA). As negative controls, a 400-bp fragment from the *mtr* coding region amplified with primers Control-F and Control-R was used instead of the 400-bp *mtr* promoter, and BSA was included instead of His_6_-SigH in the binding assays.

### Statistical analysis

Data were analysed using statistical functions in the program GraphPad Prism 5 and are shown as the mean ± standard deviation (SD). Student’s *t*-tests were performed to determine significant differences in sample means with a cut-off of P < 0.05 or P < 0.01.

## Additional Information

**How to cite this article**: Si, M. *et al*. Overexpression of Mycothiol Disulfide Reductase Enhances *Corynebacterium glutamicum* Robustness by Modulating Cellular Redox Homeostasis and Antioxidant Proteins under Oxidative Stress. *Sci. Rep*. **6**, 29491; doi: 10.1038/srep29491 (2016).

## Supplementary Material

Supplementary Information

## Figures and Tables

**Figure 1:**
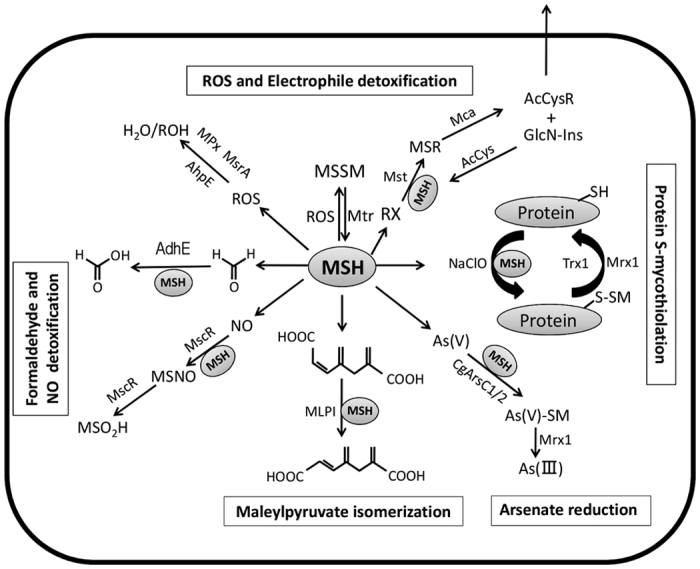
The functions of mycothiol. Mycothiol (MSH) is oxidised by reactive oxygen species (ROS) to mycothiol disulfide (MSSM). MSSM is reduced into MSH by the mycothiol disulfide reductase Mtr. MSH-dependent peroxidases, such as MPx, MsrA, and AhpE function in ROS detoxification. Electrophiles (RX) are conjugated to MSH to form MS-electrophiles (MSR), which are cleaved by the MSH S-conjugate amidase Mca to mercapturic acids (AcCysR) and exported from the cell. MSH serves as a cofactor for the alcohol dehydrogenase MscR and formaldehyde dehydrogenase AdhE for detoxification of NO and formaldehyde. Arsenate reductases CgArsC1/CgArsC2 conjugate MSH and arsenate As (V) to form As (V)-SM that is reduced to As (III) by mycoredoxin 1 (Mrx1). Metabolic reactions are catalyzed by enzymes such as maleylpyruvate isomerase requiring mycothiol as a cofactor for growth on diverse carbon sources. Under stress conditions, proteins are oxidised to mixed disulfides with MSH to form *S*-mycothiolated proteins that is reversed by the Mrx1/Mtr/MSH pathway.
