# Supplementary material for: Overexpression of Mycothiol Disulfide Reductase Enhances Corynebacterium glutamicum Robustness by Modulating Cellular Redox Homeostasis and Antioxidant Proteins under Oxidative Stress
Source: Sci Rep. 2016 Jul 7;6:29491. doi: 10.1038/srep29491 (PMC4935862; doi:10.1038/srep29491)
Supplement: Supplementary Information [file srep29491-s1.doc]

**Supplementary Information**

**Overexpression of Mycothiol Disulfide Reductase Enhances *Corynebacterium glutamicum* Robustness by Modulating Cellular Redox Homeostasis and Antioxidant Proteins under Oxidative Stress**

**Meiru Si ^a,b,c#^, Chao Zhao^a#^, Bing Zhang^a^, Dawei Wei^a^, Keqi Chen^a^, Xu Yang^a^, He Xiao^a^, Xihui Shen^a^***

^a^ State Key Laboratory of Crop Stress Biology for Arid Areas and College of Life Sciences, Northwest A&F University, Yangling, Shaanxi 712100, China;

^b^ College of Plant Protection, Northwest A&F University, Yangling, Shaanxi 712100, China;

^c^ College of Life Sciences, Qufu Normal University, Qufu, Shandong 273165, China

**Running title:** Mtr protects *C. glutamicum* against oxidative stresses

^#^ These authors contributed equally to this work.

*** For correspondence: [xihuishen@nwsuaf.edu.cn](mailto:xihuishen@nwsuaf.edu.cn)

Tel. (+86) 29 8708 1062;

Fax. (+86) 29 8709 2087

**Key words:** *Corynebacterium glutamicum*, mycothiol disulfide reductase, stress tolerance, antioxidant enzymes, redox homeostasis

**Supplementary Method**

**Western Blot Analysis**

Protein samples resolved by SDS-PAGE were transferred onto polyvinylidene fluoride (PVDF) or nitrocellulose (NC) membranes. After blocking with 4% milk powder for 2 h at room temperature, membranes were probed with the appropriate primary antibody at 4 °C overnight: anti-His, 1:1,000; anti-DNPH, 1:500. The blots were washed several times in PBST buffer (PBS buffer containing 0.2% Tween 20) and then incubated with 1:5,000 dilution of horseradish peroxidase conjugated secondary antibody (Shanghai Genomics Inc., Shanghai, China) in PBST for 1 h. The protein bands were visualized using ECL plus kit (GE Healthcare, Piscataway, NJ) following the manufacturer’s specified protocol.

**Construction of chromosomal fusion reporter strains and β-galactosidase activity assay**

The *lac*Z fusion reporter plasmid pK18*mobsacB-P_mtr_*::*lac*Z was transformed into WT(pXMJ19), Δ*sigH*(pXMJ19), and Δ*sigH*(pXMJ19-*sigH*) strains by electroporation, and the chromosomal pK18*mobsacB-P_mtr_*::*lac*Z fusion reporter strain was selected by plating onto LB-kanamycin plates. The resulting strains were grown in LB medium to an *A*_600_ of 0.9–1.0 and then treated with various stressors at the indicated concentrations at 30°C for 30 min. β-galactosidase activity was assayed using *o*-nitrophenyl-β-galactoside (ONPG) as the substrate ^1^.

**Quantitative reverse transcription (qRT)-PCR analysis**

Total RNA was isolated from exponentially growing *C. glutamicum* strains exposed to various stressors at the indicated concentrations for 30 min using the RNeasy Mini Kit (Qiagen, Hilden, Germany), along with the DNase I Kit. Purified RNA was reverse-transcribed with random 9-mer primers and M-MLV Reverse Transcriptase (TaKaRa, Dalian, China). qRT-PCR analysis was performed using the 7500 Fast Real-Time PCR System (Applied Biosystems, Foster City, CA) as described previously ^2^. The primers used are listed in Table S1. To standardize the results, the relative abundance of 16S rRNA was used as an internal standard.

***In vitro S-*mycothiolation of Prx**

*S-*mycothiolation of Prx was performed *in vitro* according to the method of Chi *et al.* ^3^. Prx was incubated with a 6 M excess of reduced MSH prior to the addition of 1 mM H_2_O_2_. After an incubation of 30 min, the sample was loaded on a Ni-NTA His⋅Bind resin-containing column (Novagen, Wisconsin, USA) and MSH and H_2_O_2_ were removed by washing (50 mM HEPES, pH 8.0, 500 mM NaCl). *S*-mycothiolated Prx was eluted in the same buffer containing 300 mM imidazole.

**MSH purification**

MSH was purified from *C. glutamicum* RES167 with thiopropyl sepharose 6B followed by Sephadex LH-20 chromatography as described ^4^. The concentration of purified MSH was measured by using the thiol-specific fluorescent-labeling HPLC method ^5^ with commercial glutathione (GSH) as the thiol standard reference. The HPLC used in this study was equipped with an Extend-C18 column (ZORBAX, 250×4.6 mm) and was operated with aqueous acetic acid-methanol gradient elution (eluant flow rate of 0.9 ml/min). The bimane derivative of MSH was eluted at about 15 min in this system.

**Table S1. Bacterial strains, plasmids, and Primers** used in this study.

| **Strains, plasmids, or primers** | **Relevant genotype description** | **References** |
| --- | --- | --- |
| **Strains** | | |
| ***Corynebacterium glutamicum*** | | |
| RES167 | Restriction-deficient mutant of ATCC13032, Δ(*cglIM-cglIR-cglIIR*) | 6 |
| Δ*sigH* | *sigH* deleted in RES167 | 7 |
| Δ*mshC* | *mshC* deleted in RES167 | 8 |
| WT(pXMJ19) | RES167 containing pXMJ19 vector | This study |
| WT(pXMJ19-His_6_-*mtr*) | Overexpression of *mtr* in RES167 | This study |
| Δ*sigH*(pXMJ19) | Δ*sigH* mutant containing pXMJ19 vector | 7 |
| Δ*sigH*(pXMJ19-*sigH*) | Complement of *sigH* in Δ*sigH* mutant | 7 |
| Δ*mshC* (pXMJ19) | *mshC* mutant containing pXMJ19 vector | 8 |
| Δ*mshC* (pXMJ19-His_6_-*mtr*) | Overexpression of *mtr* in Δ*mshC* | This study |
| ***E. coli*** | | |
| BL21(DE3) | *E. coli* expression host, *hsdS gal* (*λc*I*ts*857 *ind-l* *Sam7 nin-*5 *lac UV5-*T7 gene 1) | Novagen |
| JM109 | *recA1 supE44 endA1 hsdR17 gyrA96 relA1 thi* Δ(*lac-proAB*)F′(*traD36 proABlacI*^q^ *lacΔZM15*) | Stratagene |
| BL21(DE3)(pET28a-*mdmpi*) | Expression of MDMPI in BL21(DE3) | 9 |
| BL21(DE3)(pET28a-*fph*) | Expression of FPH in BL21(DE3) | 9 |
| BL21(DE3)(pET28a- *g12d*) | Expression of G12D in BL21(DE3) | 9 |
| BL21(DE3)(pET28a- *trxR*) | Expression of TrxR in BL21(DE3) | 10 |
| BL21(DE3)(pET28a- *trx1*) | Expression of Trx1 in BL21(DE3) | 10 |
| BL21(DE3)(pET28a- *prx*) | Expression of Prx in BL21(DE3) | 11 |
| **Plasmids** | | |
| pK18*mobsacB* | Suicide vector, Km^r^ | 12 |
| pK18*mobsacB*-Δ*mtr* | Construct used for in-frame deletion of *mtr* | This study |
| pK18*mobsacB-P_mtr_::lacZ* | *P_mtr_::lacZ* fusion in pK18*mobsacB* | This study |
| pXMJ19 | Shuttle vector (*P_tac_ lacI^q^ pBL1 oriV_C. glutamicum_* pK18 *oriV_E. coli_*) | 13 |
| pXMJ19-*sigH* | *sigH* cloned into pXMJ19 for complementation | 7 |
| pXMJ19-His_6_ | Modified pXMJ19 containing N-terminal hexahistidine tag | 9 |
| pXMJ19-His_6_-*mtr* | *mtr* cloned into pXMJ19-His_6_ for overexpression | This study |
| pET28a | Expression vector with N-terminal hexahistidine affinity tag | Novagen |
| pET28a*-sigH* | *sigH* in pET28a | 7 |
| pET28a*-mtr* | *mtr* in pET28a | 14 |
| pET28a*-mrx1:C15S* | *mrx1:C15S* in pET28a | 14 |
| pET28a*-prx* | *prx* in pET28a | 11 |
| **Primiers** | **5’-3’ sequence** |  |
| C*mt*-F | CGCGAAGCTTATGTCTGAGCAGCCAGCTTCC (HindⅢ) | To generate pXMJ19-His_6_-*mtr* |
| C*mtr*-R | ACGCGTCGAC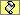CTAAAACTCTAGCCCCAGAAG (SalI) |  |
| DmtrupF | CGCGGATCCGAGCTAGTCCCCGTCGCCC(BamHI) |  |
| DmtrupR | AGTTCCGCCGAAAGCACCC |  |
| DmtrdownF | *GGGTGCTTTCGGCGGAACT*TCGAAAACG  CTCTTCTGGGGC |  |
| DmtrdownR | ACGCGTCGAC ACGCCACCCAAGGTGATTGCAG (SalI) |  |
| *P_mtr_*-F1 | TCCCCCGGGGGGTTCACGGCATGAC (SmaI) | To generate pK18*mobsacB-P_mtr_::lacZ* |
| *P_mtr_*-R | TCTAGACTGCTCAGACATAAGATG (XbaI) |  |
| *P_mtr_*-F2 | CCAACCGTCGAGGGAAATG | To generate the 400 bp *mtr* promoter probe |
| lacZY-F | CATCTTATGTCTGAGCAGTCTAGAACTAGT ATGACCATGATTACGGATTC(SpeI) |  |
| lacZY-R | AAAACTGCAGTTAAGCGACTTCATTCACCTG(PstI) |  |
| Prx-F | CGCGGATCCATGGGGTCCATGGCTAAAACAC (BamHI) | For cloning *prx* into pET28a |
| Prx-R | ACGCGTCGACTTAGTTCAGCGCAGCAAGTGCAG (SalI) |  |
| Control-F | CATCGTTTGTTGATTCCA | To produce the 400 bp EMSA control DNA |
| Control-R | ATCTTCACGCCGCCGTCG |  |
| RTNcgl2502F | CGTGGCATCCAAGTGCGG | For *mpx* qRT-PCR |
| RTNcgl2502R | CGCGAAAGCACTCACCTC |  |
| RTNcgl2985F | TCCTTCTGTTCTCATTTTCA | For *trx1* qRT-PCR |
| RTNcgl2985R | GTGCTTCTCTAGTTTTTCCA |  |
| RTNcgl0808F | TAACCATTTACGCCACAGATTG | For *mrx1* qRT-PCR |
| RTNcgl0808R | GTTGACTGACTTAACCCACTCG |  |
| RTNcgl2826F | CTTCGGTTCTTTCGCTAAGTTCC | For *sod* qRT-PCR |
| RTNcgl2826R | ATGTTGCCCTGCTGGTCGGTGAG |  |
| RTNcgl0251F | TTCCTCCTCCAACCACA | For *cat* qRT-PCR |
| RTNcgl0251R | TCTGCCAATCGCTCC |  |
| RTNcgl2825F | CCGCCAGGGTAACGATGT | For *msrA* qRT-PCR |
| RTNcgl2825R | CCGATTTCCGTGGTGATTT |  |
| 16SF | AGAACCTTACCTGGGCTTGA |  |
| 16SR | CGCTCGTTGCGGGACTTA |  |

Underlined sites indicate restriction enzyme cutting sites added for cloning. Letters in italic denote the mutation sites in overlap PCR for site-directed mutation.

**
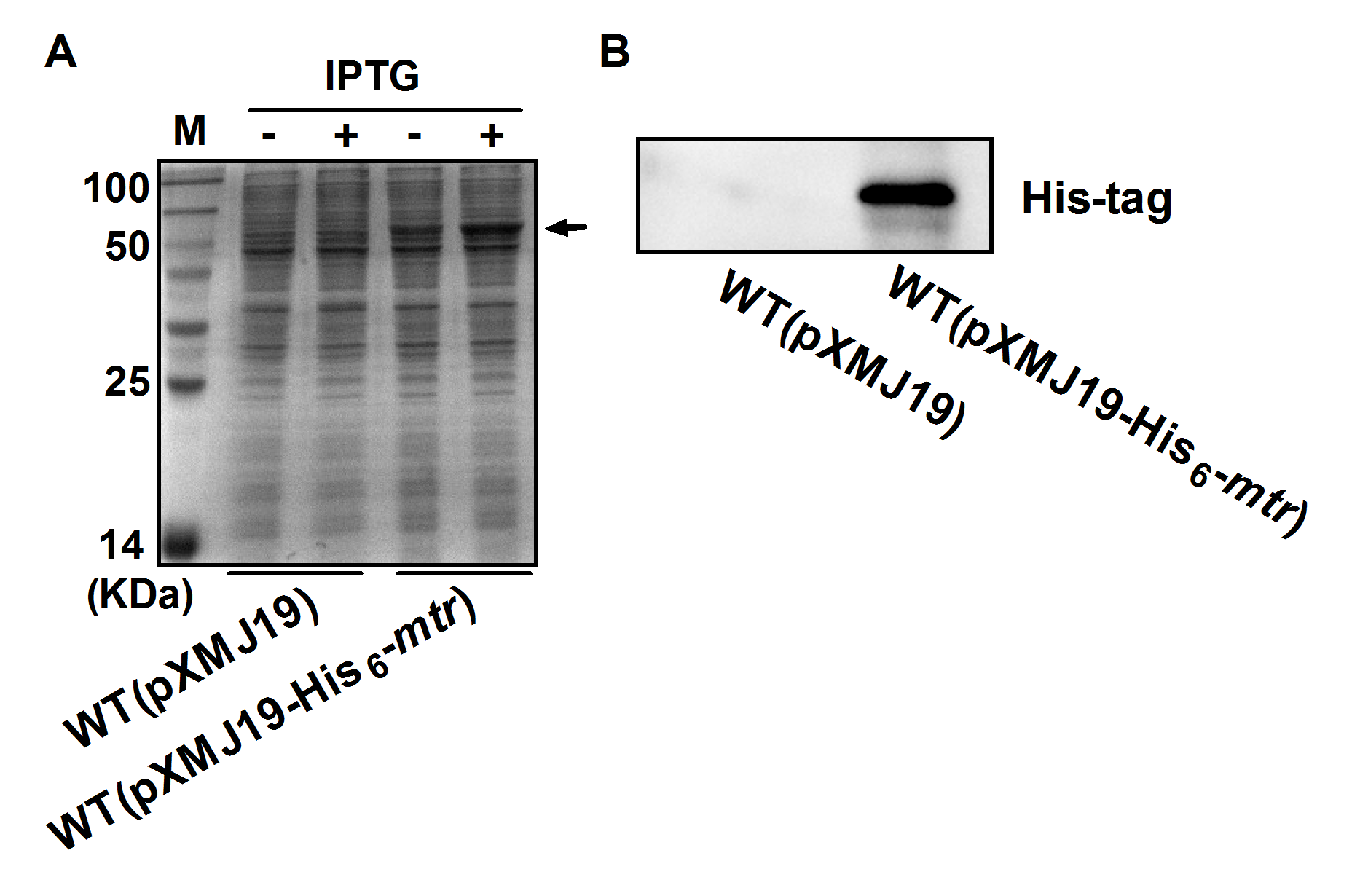
**

**Figure S1** His_6_-Mtr expression detected in WT(pXMJ19-His_6_-*mtr*) and WT(pXMJ19). (A) SDS-PAGE analysis of proteins expressed in stationary phase WT(pXMJ19-His_6_-*mtr*) and WT(pXMJ19) in the absence (-) and presence (+) of 0.5 mM IPTG. Crude extracts (20 μg) were loaded, run on 15% SDS-PAGE, and visualised by Coomassie staining. M, protein marker; arrows, His_6_-Mtr. (B) Western blot analysis of Mtr protein in stationary phase WT(pXMJ19-His_6_-*mtr*) and WT(pXMJ19) cells in the presence of 0.5 mM IPTG. Western blot analysis was carried out using an anti-His antibody.

**
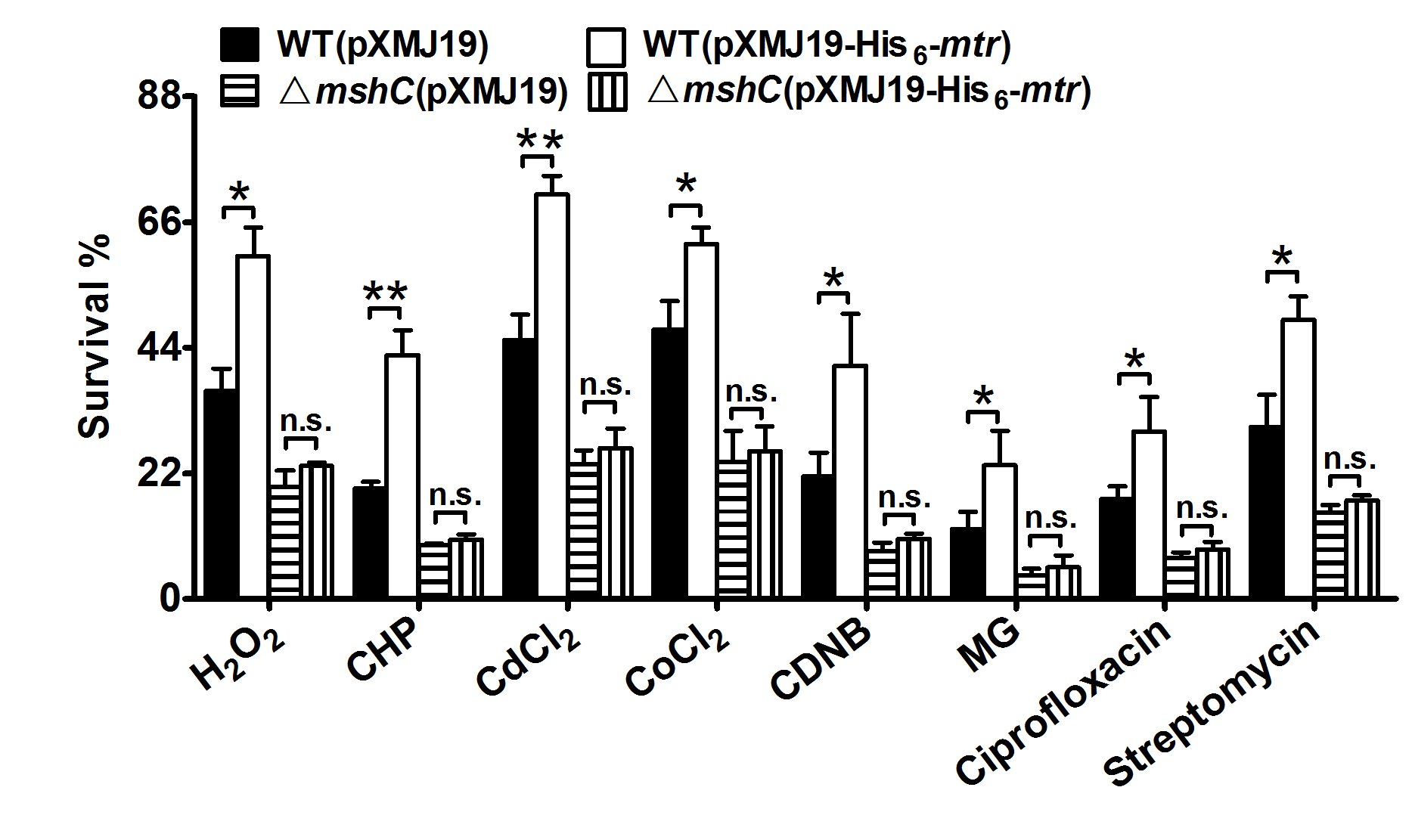
**

**Figure S2** Effects of Mtr overexpression on resistance to oxidative stresses in Δ*mshC*. Survival rates of indicated *C. glutamicum* strains after being challenged with multiple oxidative stresses for 30 min. Mean values with standard deviations (error bars) from at least three independent experiments are shown. n.s.: not significant *: P ≤ 0.05. **: P ≤ 0.01.

**Figure S3** The correlation between MSH:MSSM ratio and higher resistance. Survival rates of WT(pXMJ19-His_6_-*mtr*) strain exposed to untreated, 100 mM H_2_O_2_, 11 mM CHP, 375 μg/ml ciprofloxacin, 0.3 mM CdCl_2_, 70 mM CDNB were against the MSH:MSSM ratios of WT(pXMJ19-His_6_-*mtr*) strain with the corresponding treatments to form the calibration curve.

**References**

1. Miller, J.H. *A short course in bacterial genetics: a laboratory manual* a*nd handbook for Escherichia coli and related bacteria.* (Cold Spring Harbor Laboratory Press*,* Cold Spring Harbor, New York, 1992).
2. Li, M. *et al*. Identification of novel targets of cyanobacterial glutaredoxin. *Arch. Biochem. Biophys.* **458**, 220–228 (2007).
3. Chi, B.K. *et al*. Protein *S*-mycothiolation functions as redox-switch and thiol protection mechanism in *Corynebacterium glutamicum* under hypochlorite stress. *Antioxid. Redox Signal.* **20**, 589–605 (2014).
4. Feng, J. *et al*. The gene *ncgl2918* encodes a novel maleylpyruvate isomerase that needs mycothiol as cofactor and links mycothiol biosynthesis and gentisate assimilation in *Corynebacterium glutamicum.* *J. Biol. Chem.* **281**, 10778–10785 (2006).
5. Newton, G.L. *et al*. Distribution of thiols in microorganisms: mycothiol is a major thiol in most actinomycetes. *J. Bacteriol*. **178**, 1990-1995 (1996).
6. Tauch, A. *et al*. Efficient electrotransformation of *Corynebacterium diphtheriae* with a mini-replicon derived from the plasmid pGA1. *Curr. Microbiol.* **45**, 362–367 (2002).
7. Si, M.R. *et al*. Functional characterization of *Corynebacterium glutamicum* mycothiol S-conjugate amidase. *PLoS One*, **9**, e115075 (2014).
8. Liu, Y.B. *et al*. Physiological roles of mycothiol in detoxification and tolerance to multiple poisonous chemicals in *Corynebacterium glutamicum*. *Arch. Microbiol.* **195**, 419-429 (2013).
9. Liu, Y.B. *et al*. Enhancing *Corynebacterium glutamicum* robustness by overexpressing a gene, *mshA*, for mycothiol glycosyltransferase. *Biotechnol. Lett.* **36**, 1453-1459 (2014).
10. Si, M.R. *et al*. Functional characterization of a mycothiol peroxidase in *Corynebacterium glutamicum* that uses both mycoredoxin and thioredoxin reducing systems in the response to oxidative stress. *Biochem. J.* **469**, 45-57 (2015).
11. Si, M.R. *et al*. NrdH-redoxin enhances resistance to multiple oxidative stresses by acting as a peroxidase cofactor in *Corynebacterium glutamicum*. *Appl. Environ. Microbiol.* **80**, 1750–1762 (2014).
12. Schäfer, A., Tauch., A, Jager, W., Kalinowshi, J., Thierbach, G. & Pühler, A. Small mobilizable multi-purpose cloning vectors derived from the *Escherichia coli* plasmids pK18 and pK19: selection of defined deletions in the chromosome of *Corynebacterium glutamicum*. *Gene*. **145**, 69-73 (1994).
13. Jakoby, M., Ngouoto-Nkili, C.E. & Burkovski, A. Construction and application of new *Corynebacterium glutamicum* vectors. *Biotechno. Techniques* **13**, 437–441 (1999).
14. Si, M.R. *et al*. *Corynebacterium glutamicum* methionine sulfoxide reductase A uses both mycoredoxin and thioredoxin for regeneration and oxidative stress resistance. *Appl. Environ. Microbiol.* **81**, 2781-2796 (2015).
